# Impaired Glymphatic Clearance, Measured Using Diffusion Tensor Image Analysis Along the Perivascular Space (DTI‐ALPS), is Linked to Poor Cognitive Outcomes in Parkinson's Disease

**DOI:** 10.1002/mds.30325

**Published:** 2025-08-07

**Authors:** Angeliki Zarkali, George Thomas, Ross Paterson, Naomi Hannaway, Ivelina Dobreva, Amanda J. Heslegrave, Elena Veleva, Henrik Zetterberg, Rimona S. Weil

**Affiliations:** ^1^ Dementia Research Centre, Institute of Neurology University College London London UK; ^2^ National Hospital for Neurology and Neurosurgery Queen Square London UK; ^3^ UK DRI Fluid Biomarker Lab and Biomarker Factory University College London London UK; ^4^ Movement Disorders Centre, Institute of Neurology University College London London UK; ^5^ Department of Imaging Neuroscience, Institute of Neurology University College London London UK

**Keywords:** glymphatic clearance, white matter degeneration, Parkinson's disease, Parkinson's dementia, iron accumulation, cortical thickness

## Abstract

**Background:**

Impaired glymphatic clearance may contribute to pathological accumulations in Parkinson's (PD), but how it interacts with other processes causing dementia remains unclear. Diffusion tensor image analysis along the perivascular space (DTI‐ALPS) has been proposed as an indirect proxy for glymphatic clearance.

**Objectives:**

To clarify DTI‐ALPS' relationship with cognition in PD, and its relationship with established imaging markers.

**Methods:**

We assessed DTI‐ALPS in 98 PD patients (31 PD poor outcomes: dementia, mild cognitive impairment, frailty, or death within a 3‐year follow‐up; 67 PD good outcomes) and 28 controls. We assessed DTI‐ALPS' relationship to cognition, white matter (fiber cross‐section), cortical thickness, iron accumulation (quantitative susceptibility mapping [QSM]), and plasma markers [phosphorylated tau‐181 (*p*‐tau181 and neurofilament light (NfL)] cross‐sectionally and longitudinally).

**Results:**

DTI‐ALPS was lower in PD‐poor outcomes compared with PD good outcomes and controls (*P* = 0.005) with further longitudinal reductions only in PD poor outcomes (group × time interaction: *β* = −0.013, *P* = 0.021). Lower DTI‐ALPS was associated with lower fiber cross‐section in PD, at baseline and longitudinally but with different spatial distribution from white matter changes relating to PD cognition. There was no correlation between baseline DTI‐ALPS and plasma *p*‐tau181 (*P* = 0.642), NFL (*P* = 0.448), or baseline cortical thickness. Lower DTI‐ALPS was associated with accelerated cortical thinning within left precentral gyrus and changes in brain iron distribution.

**Conclusions:**

PD patients who develop poor outcomes show lower DTI‐ALPS, potentially reflecting impaired glymphatic clearance. DTI‐ALPS correlated with white matter integrity and brain iron accumulation. However, both showed different spatial distribution than that seen in PD dementia, suggesting DTI‐ALPS captures a distinct contribution to cognitive decline. © 2025 The Author(s). *Movement Disorders* published by Wiley Periodicals LLC on behalf of International Parkinson and Movement Disorder Society.

Cognitive impairment is common in Parkinson's disease (PD), with dementia developing in nearly half of PD patients within 10 years^1^ with high personal, societal, and financial burden.^2^, ^3^ PD dementia is multifactorial: outside of dopaminergic degeneration and alpha‐synuclein accumulation, other processes including white matter degeneration,^4^, ^5^ iron accumulation,^6^ beta‐amyloid, tau co‐pathology,^7^ and cerebrovascular disease^8^ contribute to the development and progression of cognitive decline.

Additionally, recent studies have implicated glymphatic system dysfunction in pathophysiology of neurodegenerative disorders.^9^ The glymphatic system, first proposed by Iliff et al.,^10^ is a network of perivascular channels facilitating cerebrospinal fluid (CSF) flow through the brain and plays a key role in clearance of soluble waste through astrocytic aquaporin‐4 channels.^10^, ^11^ In mouse models, monomeric alpha‐synuclein propagates to remote brain regions via the glymphatic system,^12^ and suppression of glymphatic activity via aquaporin‐4 gene deletion or acetazolamide administration leads to increased alpha‐synuclein accumulation.^13^ Similarly, impaired glymphatic clearance is seen in mouse tauopathy models^14^ and leads to beta‐amyloid accumulation and cognitive deficits in mice.^15^

Techniques to assess the glymphatic system in vivo have recently been developed; however, most are invasive, requiring tracer injection and sequential imaging.^16^ Amongst the non‐invasive methods is diffusion tensor image analysis along the perivascular space (DTI‐ALPS). This is thought to reflect glymphatic clearance by measuring diffusivity of water molecules in the perivascular space at the level of the body of the lateral ventricle.^17^ Although an indirect measure from a single brain region aimed to reflect whole‐brain clearance, DTI‐ALPS strongly correlates with gold‐standard measures of glymphatic function^18^ and conventional magnetic resonance imaging (MRI) markers of cerebrovascular disease.^19^ Additionally, DTI‐ALPS recently showed positive correlations with sleep quality, younger age, and better cognition in health,^20^ in accordance with what would be expected from a metric of glymphatic clearance.

Several studies have shown reduced DTI‐ALPS index in PD,^21^ including in idiopathic rapid eye movement (REM) sleep behavior disorder, reflecting prodromal synucleinopathy.^22^ DTI‐ALPS is inversely correlated with motor symptoms and disease severity^23^, ^24^ and linked to faster motor and cognitive decline in PD.^24^ How and when impaired glymphatic clearance impacts other processes contributing to PD dementia is not clear; neither is the interrelationship between DTI‐ALPS and other imaging metrics that change with cognitive impairment in PD.^25^

However, there is evidence that these may interact. In Alzheimer's disease (AD), where DTI‐ALPS is also reduced and correlates with cognition,^17^, ^26^, ^27^, ^28^ DTI‐ALPS is linked to beta‐amyloid^28^ and tau‐PET burden.^27^, ^28^ It also correlates with grey matter atrophy, which may mediate its effect on cognition.^27^, ^28^, ^29^ In healthy older adults, lower DTI‐ALPS correlates with lower grey matter volume,^30^ and relates to iron deposition within the basal ganglia,^31^ assessed using quantitative susceptibility mapping (QSM). In PD, co‐pathologies are particularly prevalent,^32^ with high heterogeneity in cognitive presentation and underlying pathology and an increased interest in biomarkers for risk‐stratification and disease progression.^25^ We have previously shown in PD that cognitive decline is linked with reductions in fiber cross‐section^4^, ^33^ and alterations in QSM signal.^6^, ^34^ Understanding the relationship between biomarkers of pathological protein accumulation, tissue changes such as iron build‐up, small vessel disease, and glymphatic clearance may yield important insights into their role in the development of cognitive impairment in PD.

Here, we used DTI‐ALPS, proposed as an indirect measure of glymphatic clearance, and assessed its ability to predict poor outcomes (development of dementia, mild cognitive impairment [MCI], frailty or death over 3 years) in 98 PD patients and 28 controls. We examined the relationship between DTI‐ALPS and cognitive performance at baseline and longitudinally in these patients. Additionally, we assessed how DTI‐ALPS relates to other processes that we have previously shown in this cohort to be correlated with poor cognitive outcomes in PD cross‐sectionally and longitudinally.^4^, ^6^, ^33^, ^34^ Specifically, in patients with PD we examined DTI‐ALPS' relationship with: (1) white matter macrostructural integrity, assessed using whole‐brain fiber cross‐section and plasma neurofilament light chain (NfL); (2) grey matter atrophy, using cortical thickness; (3) iron accumulation, using QSM; and (4) beta‐amyloid and tau accumulation, using plasma levels of phosphorylated tau at threonine 181 (*p*‐tau181).

## 1 Methods

### 1.1 Participants

We included 98 PD patients and 28 controls from the Vision in Parkinson's Disease study, University College London (UCL), London, UK; this has been previously detailed.^4^, ^35^ PD patients fulfilled International Parkinson and Movement Disorder Society (MDS) clinical diagnostic criteria.^36^ PD patients with a diagnosis of Parkinson's dementia (PDD) or mild cognitive impairment (PD‐MCI) were excluded. Participants were assessed at baseline, and after 18 months (Session 2) and 36 months (Session 3). All participants provided written informed consent; the study was approved by the Queen Square Research Ethics Committee.

Neuropsychology assessments included two general measures of cognition (Mini‐Mental State Examination^37^ [MMSE] and Montreal Cognitive Assessment^38^ [MoCA]), and two tests per cognitive domain.^35^ A composite cognitive score was calculated as the averaged *z*‐scores of each participant's MoCA plus one task per cognitive domain (Inverted Stroop Colour Naming Time, Category Fluency, Letter Fluency, Recognition Memory Test, and Hooper Visual Organization Test).^35^ Motor function was assessed using International Parkinson and Movement Disorder Society‐Unified Parkinson's Disease Rating Scale (MDS‐UPDRS)^39^ and Timed Up and Go (TUG) test^40^. Levodopa equivalent daily doses (LEDD) were calculated.^41^

As previously,^4^, ^35^ we defined poor cognitive outcomes in PD if any of the following occurred during follow‐up: death, frailty, dementia, or PD‐MCI, defined as persistent performance below 1.5 standard deviations in two tests in one cognitive domain or one test in two cognitive domains.^42^ The definition of poor outcomes was chosen based on our previous work examining imaging predictors^4^, ^34^, ^35^ and reflecting real‐world poor outcomes.

### 1.2 MRI Acquisition

MRI data for all sessions and participants were acquired on the same 3 T Siemens Prisma scanner (Siemens) with a 64‐channel coil and parameters: *diffusion weighted imaging (DWI)*: b0 (AP‐ and PA‐directions), *b* = 50s/mm^2^ 17 directions, *b* = 300 s/mm^2^ 8 directions, *b* = 1000s/mm^2^ 64 directions, *b* = 2000s/mm^2^ 64 directions, 2 mm^3^ isotropic voxels, TE = 3260 ms, TR = 58 ms, 72 slices, acceleration factor = 2; *3D magnetization prepared rapid acquisition gradient echo (MPRAGE; T*_*1*_‐*weighted imaging*): 1 mm^3^ isotropic voxels, TE = 3.34 ms, TR = 2530 ms, flip angle = 7°; *susceptibility weighted imaging (SWI; used for QSM)*: 1 m^3^ isotropic voxels, 3D flow‐compensated spoiled‐gradient‐recalled echo sequence, flip angle = 12°, TE = 18 ms, TR = 25 ms, receiver bandwidth = 110 Hz/pixel, matrix size = 204 × 224 × 160.

### 1.3 MRI Quality Control and Preprocessing

Raw volumes were visually inspected and excluded if artefact was present. Images passing quality assurance underwent preprocessing using established pipelines.^4^, ^34^

Briefly, for *white matter imaging*, DWI images were preprocessed using the Mrtrix3 pipeline including denoizing,^43^ Gibbs artefact removal,^44^ eddy‐current, motion,^45^ and bias field correction^46^; images were up‐sampled (1.3 mm^3^) and intensity normalization performed. Multi‐shell three‐tissue constrained spherical deconvolution^47^ was performed and fiber‐orientation distributions calculated per participant and registered to a group‐averaged template.^48^ Fiber cross‐section was derived per participant; this was chosen as it is a sensitive metric of white matter integrity loss related to poor outcomes in PD^4^, ^33^, ^49^ and reflects pathological protein accumulation and subsequent neurodegeneration, rather than cerebrovascular changes.^50^

For *grey matter imaging*, T_1_‐weighted images were processed with default cross‐sectional parameters using FreeSurfer6.0, then the longitudinal pipeline,^51^ manually correcting and reprocessing surface reconstructions where necessary.

For *QSM*, 3D‐complex phase data were unwrapped from SWI images using rapid path‐based minimum spanning tree algorithm (ROMEO)^52^ and brain masks calculated using Brain Extraction Tool (BET).^53^ Background field removal was performed using Laplacian boundary value extraction^54^ and dipole inversion was performed using multi‐scale dipole inversion^55^ to derive susceptibility maps.

### 1.4 DTI‐ALPS Index Calculation

DTI‐ALPS was calculated in Mrtrix3 using custom scrips based on Taoka et al.^17^ DTI‐ALPS is calculated from the mean diffusivity of specific regions at the level of the body of the lateral ventricle. It is based on the assumption that movement of interstitial fluid in the perivascular space at the level of the body of the lateral ventricle is dominant parallel to the medullary veins, which run perpendicular to the ventricular wall; x‐axis (right–left direction). The y‐axis (rostral–caudal) direction contains the projection fibers, and z‐axis (anterior–posterior) association fibers, orthogonal to the perivascular space direction.^17^ DTI‐ALPS index is defined as:

$$\mathrm{DTI}‐\text{ALPS}=\frac{\text{mean}\;\left(D_{\text{xxproj}},D_{\text{xxassoc}}\right)}{\text{mean}\;\left(D_{\text{yyproj}},D_{\text{zzassoc}}\right)}$$

where *D*_*xxproj*_ is mean diffusivity of projections fibers in the x‐axis, *D*_*xxassoc*_ mean diffusivity of association fibers in the x‐axis, *D*_*yyproj*_ mean diffusivity of projection fibers in the y‐axis, and *D*_*zzassoc*_ mean diffusivity of association fibers in the z‐axis.^17^

We used an automated atlas‐based approach to identify projection and association fibers based on a pipeline with good inter‐rater, inter‐scanner, and test–retest reliability.^56^ Projection fibers were defined using the superior corona radiata (SCR) in the Johns Hopkins Atlas of Human Functional Anatomy (JHU) (center coordinates left [116, 110, 99], right [64, 110, 99]) and association fibers using the superior longitudinal fasciculus (SLF; left [128, 110, 99], right [51, 110, 99]).

The DTI‐ALPS index was calculated separately for left and right hemispheres and a mean index calculated as the average of the two. As DTI‐ALPS between hemispheres was highly intercorrelated (Spearman rho = 0.819, *P* < 0.001) we report the mean index, referred hereby as DTI‐ALPS index. Lower DTI‐ALPS index reflects lower diffusivity and is proposed to reflect worse overall glymphatic clearance.^17^

### 1.5 Plasma Biomarkers

Plasma samples were collected at Session 2; results were available for 87 participants for *p*‐tau181 and 88 participants for NfL. A total of 13 participants had plasma collected at Session 3 due to COVID‐19 lockdown restrictions during the Session 2 testing period. *p*‐tau181 was measured using the Simoa *p*‐tau181 Advantage Kit, and NfL using the tSimoa Human Neurology 4‐Plex A (N4PA) assay (Quanterix); analysts were blinded to clinical data. All measurements were above the assay limit of detection. Quality control samples had mean intra‐assay and inter‐assay coefficient of variation <10%.

### 1.6 Statistical Analysis

Group differences were assessed using ANOVA (post‐hoc Dunn) for normally distributed and Kruskal–Willis (post‐hoc Tukey) for non‐normally distributed variables. To assess DTI‐ALPS’ relationship to clinical and imaging metrics we used partial Spearman correlation cross‐sectionally, and repeated‐measures ANOVA longitudinally, correcting for age and sex.

To investigate the relationship *between glymphatic function and white matter degeneration* we performed whole‐brain fixel‐based analysis of fiber cross‐section. Whole‐brain probabilistic tractography was performed on the population template (20 million streamlines), filtered to 2 million using spherical deconvolution‐informed filtering of tractograms (SIFT).^57^ Connectivity‐based fixel‐enhancement was performed on white matter fixels of the JHU Atlas^58^ (5000 permutations, family‐wise error (FWE)‐corrected *P*‐value *q* < 0.05 and 10 voxels extent‐based threshold). As DTI‐ALPS is derived from DWI images, to minimize bias we used a fixel‐based metric (fiber cross‐section), derived using a higher tensor model rather than DTI. We also used left DTI‐ALPS index to correlate with right hemisphere fixels and right DTI‐ALPS index for left hemisphere fixels to minimize bias within the specific regions from which DTI‐ALPS is derived. We report combined results. The following analyses were performed: (1) baseline fiber cross‐section against baseline DTI‐ALPS (age, sex and total intracranial volume as covariates) and (2) follow‐up fiber cross‐section against baseline DTI‐ALPS (age, sex, intracranial volume, and time‐between‐scans as covariates).

To investigate the relationship *between glymphatic function and cortical thickness* we used general linear models in Freesurfer. The following analyses were performed with the same nuisance covariates as mentioned above: (1) baseline DTI‐ALPS against baseline cortical thickness and (2) baseline DTI‐ALPS against longitudinal cortical thickness. Significance maps were corrected for multiple comparisons using false discovery rate (FDR) combined over left and right hemispheres.

To relate *DTI‐ALPS to QSM signal* we first performed spatial standardization of susceptibility maps using an average T_1_‐weighted template.^34^ Each participant's bias‐corrected magnitude image was registered (rigid and affine) to the respective MPRAGE. QSM images were transformed to template space through high‐order b‐spline interpolation. Images were smoothed using a 3D Gaussian kernel (3 mm standard deviation) and smoothing‐compensated. Whole‐brain QSM analysis was performed using permutation with threshold‐free cluster enhancement (fslrandomise, 10,000 permutations, FWE‐corrected *q* < 0.05). The following analyses were performed with the same nuisance covariates: (1) baseline susceptibility against baseline DTI‐ALPS and (2) follow‐up susceptibility against baseline DTI‐ALPS.

All whole‐brain analyses were performed within all PD patients as we wanted to assess DTI‐ALPS relationship with other imaging metrics associated with cognitive decline in the context of PD. Due to the number of PD poor outcomes, we were not adequately powered for whole‐brain correlation analyses within subgroups.

To explore the interplay between DTI‐ALPS, white and grey matter degeneration, iron accumulation, and poor cognitive outcomes in PD, we conducted mediation analyses to derive total, direct, and indirect effects. These are detailed in Supplementary Material.

### 1.7 Data Sharing

Anonymized, group‐level summary data and analysis code are available on GitHub (https://github.com/AngelikaZa/AngelikaZa‐DTIALPSinPDcognitiveImpairment). Imaging and clinical data will be shared upon reasonable request to the corresponding author.

## 2 Results

A total of 98 PD participants and 28 controls were included; demographics are shown in Table 1. Of PD poor outcomes, 2 died, 6 developed frailty, 11 developed dementia (1 also developed frailty and 1 died), and 14 developed MCI.

**TABLE 1:** Study participants’ baseline demographics and clinical assessments

Parameter	HC (n = 28)	PD with good outcome (n = 67)	PD with poor outcome (n = 31)	*P*‐value
Demographic characteristics				
Age (years)	**65.7 (9.1)**	**62.4 (7.0)**	**68.5 (8.5)**	**0.004** ^b^
Sex (F/M)	**15/13**	**38/29**	**8/23**	**0.014** ^b^
Handedness (R/L)	25/3	63/4	27/4	0.516
Years in education	17.9 (2.3)	16.9 (2.6)	17.5 (2.9)	0.386
Total intracranial volume	1393.7 (95.5)	1459.7 (135.5)	1468.9 (120.5)	0.036^**ns**^
Clinical assessments				
MMSE	**29.2 (0.9)**	**29.1 (1.1)**	**28.4 (1.5)**	**0.004** ^a^, ^b^
MOCA	**28.9 (1.3)**	**28.5 (1.4)**	**26.3 (3.2)**	**0.001** ^a^, ^b^
Combined cognitive score	**0.07 (0.6)**	**− 0.01 (0.6)**	**−0.93 (0.9)**	**0.001** ^a^, ^b^
Disease duration	–	4.1 (2.3)	4.9 (3.4)	0.209
Affected side at onset (R/L/BL)	–	35/6/26	14/7/10	0.082
UPDRS total score	–	**41.4 (18.7)**	**55.6 (32.9)**	**0.007**
UPDRS motor score	–	**19.5 (9.5)**	**26.6 (18.8)**	**0.042**
LEDD	–	451.8 (271.4)	484.3 (231.7)	0.195
Sleep (RBDSQ)	–	4.1 (2.5)	4.5 (2.5)	0.224
Image quality metrics				
Coefficient of joint variation*	0.7 (0.2)	0.7 (0.2)	0.8 (0.2)	0.206
Total signal to noise ratio*	47.9 (12.1)	47.4 (14.6)	42.5 (15.2)	0.153
Entropy focus criterion	135.5 (28.0)	131.1 (25.5)	127.6 (24.4)	0.082

*Note*: All results are shown as mean (standard deviation) unless otherwise specified. Bold type denotes statistical significance.

Abbreviations: HC, healthy controls; PD, Parkinson's disease; F, female; M, male; R, right; L, left; ns, not significant; MMSE, Mini‐Mental State Examination; MOCA, Montreal Cognitive Assessment; combined cognitive score, calculated as the mean of the *z‐*scores of two cognitive scores per cognitive domain (z‐scored against control performance at baseline); BL, bilateral; UPDRS, Unified Parkinson's Disease Rating Scale; LEDD, total equivalent levodopa dose; RBDSQ, REM Sleep Behavior Disorder (RBD) Questionnaire.

^a^ Post‐hoc significant difference between HC and PD poor outcome.

^b^ Post‐hoc significant difference between PD good and PD poor outcome (also in bold).

* Lower values are better.

### 2.1 Lower Baseline DTI‐ALPS Index is Associated with Poor Outcomes in PD

**FIG. 1:**
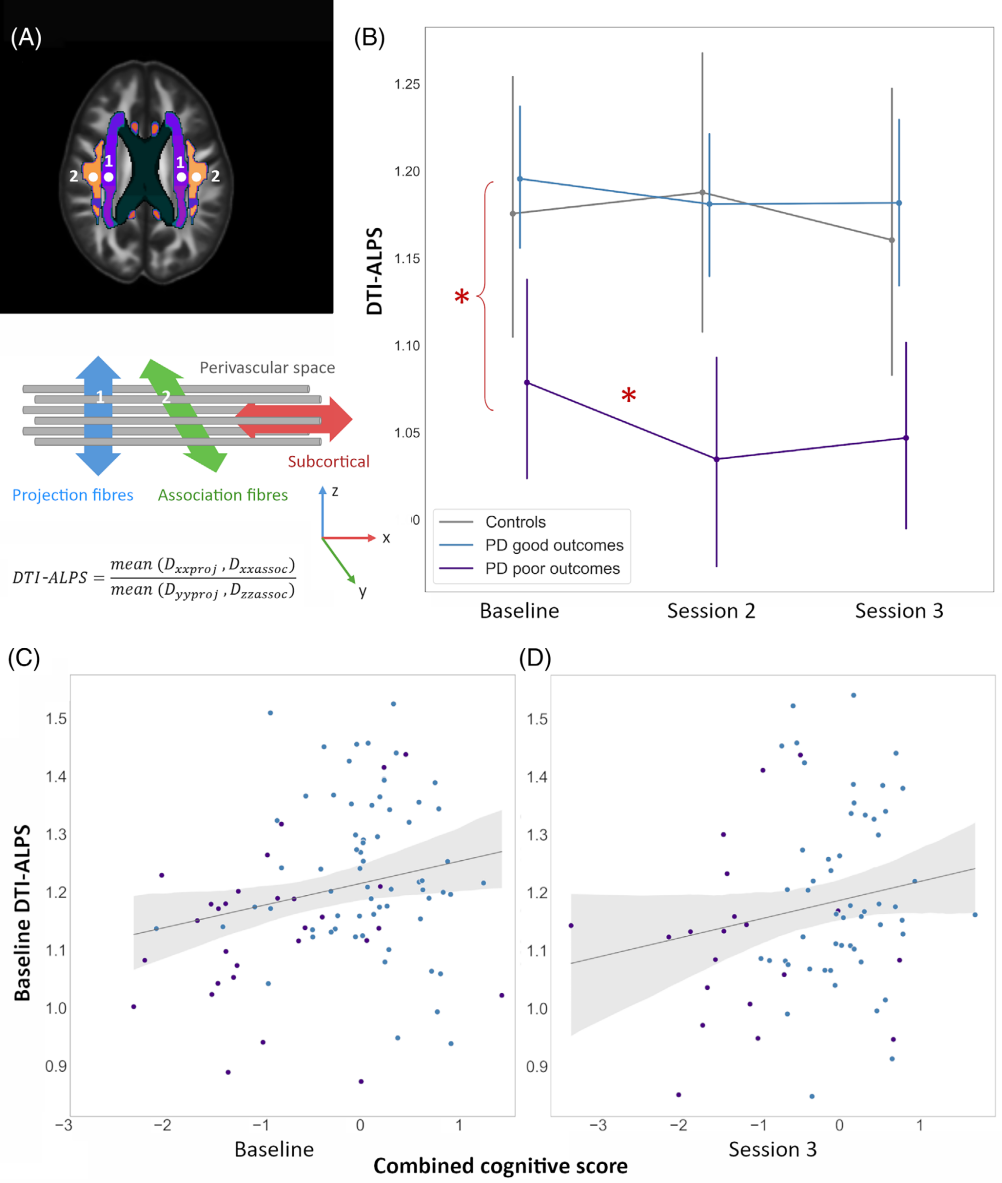
Diffusion tensor image analysis along the perivascular space (DTI‐ALPS) index is associated with poor cognitive outcomes in Parkinson's disease (PD). (A) Calculation of DTI‐ALPS. DTI‐ALPS is based on the assumption that diffusion of fluid in the perivascular space at the level of the body of the lateral ventricle is dominant parallel to the medullary veins which run along the x‐axis. Lower values indicate impaired glymphatic clearance. The diagram shows the positional relationship of the perivascular space (x‐axis), projection fibers (z‐axis), and association fibers (y‐axis). Their orthogonal relationship makes it possible to evaluate the diffusion component along the medullary vessels by eliminating the influence of the fiber component using the formula: $\mathrm{DTI}‐\text{ALPS}=\frac{\text{mean}\;\left(D_{\text{xxproj}},D_{\text{xxassoc}}\right)}{\text{mean}\;\left(D_{\text{yyproj}},D_{\text{zzassoc}}\right)}$. We identified projection (1) and association (2) fibers at the level of lateral ventricle body as 5 mm spheres within the superior corona radiata (SCR) and the superior longitudinal fasciculus (SLF) of the JHU‐ICBM‐DTI‐81‐white‐matter labelled Atlas. Region‐of‐interest (ROI) center coordinates were SCR: left (116, 110, 99) and right (64, 110, 99) and SLF: left (129, 110, 99) and right (51, 110, 99) in the JHU‐ICBM‐FA template. (B) DTI‐ALPS index is lower in patients with PD who progressed to poor outcomes. Patients with PD who had poor cognitive outcomes had lower DTI‐ALPS index (potentially indicating impaired glymphatic clearance) at baseline (*P* = 0.005) and showed additional longitudinal reductions (group × time interaction *β* = −0.057, *P* < 0.001) within 18 months follow‐up. (C) Baseline DTI‐ALPS index was correlated with cognition at baseline. Baseline DTI‐ALPS index was correlated with combined cognitive scores (*z*‐scores of Montreal Cognitive Assessment [MoCA] plus one test per cognitive domain) at baseline in patients with PD (rho = 0.226, *P* = 0.027). (D) Baseline DTI‐ALPS index was correlated with cognition at 3‐year follow‐up. Baseline DTI‐ALPS index was correlated with combined cognitive scores at Session 3 (rho = 0.25, *P* = 0.014). However, baseline DTI‐ALPS index was not correlated with longitudinal change in combined cognitive scores (*P* = 0.088). [Color figure can be viewed at wileyonlinelibrary.com]

PD poor outcomes showed lower DTI‐ALPS index compared with PD good outcomes and controls (PD poor outcomes mean ± SD = 1.08 ± 0.16 vs. PD good outcomes 1.19 ± 0.16 vs. controls 1.18 ± 0.20, *P* = 0.005) (Fig. 1B). PD poor outcomes showed further longitudinal reductions (group × time interaction *β* = −0.057, *P* < 0.001) within the 18‐month follow‐up (Fig. 1B). Baseline DTI‐ALPS index was correlated with combined cognitive scores at baseline (rho = 0.226, *P* = 0.027) (Fig. 1C) and Session 3 (rho = 0.25, *P* = 0.014) (Fig. 1D) but with no difference in combined cognitive scores between sessions (*P* = 0.088). Within individual subgroups (PD poor outcomes and PD good outcomes) DTI‐ALPS was not significantly correlated with any clinical measures, likely due to lack of power.

Having confirmed the association between DTI‐ALPS index and poor cognitive outcomes in PD, we aimed to assess how impaired glymphatic clearance (reflected by lower DTI‐ALPS) relates to processes we have previously shown to correlate with cognitive impairment in our cohort^4^, ^6^, ^33^, ^34^; specifically loss of white matter macrostructural integrity (using fiber cross‐section and plasma NfL), iron accumulation (using QSM), amyloid co‐pathology (using plasma *p*‐tau181), and grey matter atrophy (using cortical thickness).

### 2.2 Lower DTI‐ALPS Index is Associated with Changes in White Matter Macrostructure but with a Different Spatial Distribution to Changes Linked to Cognitive Impairment in PD

**FIG. 2:**
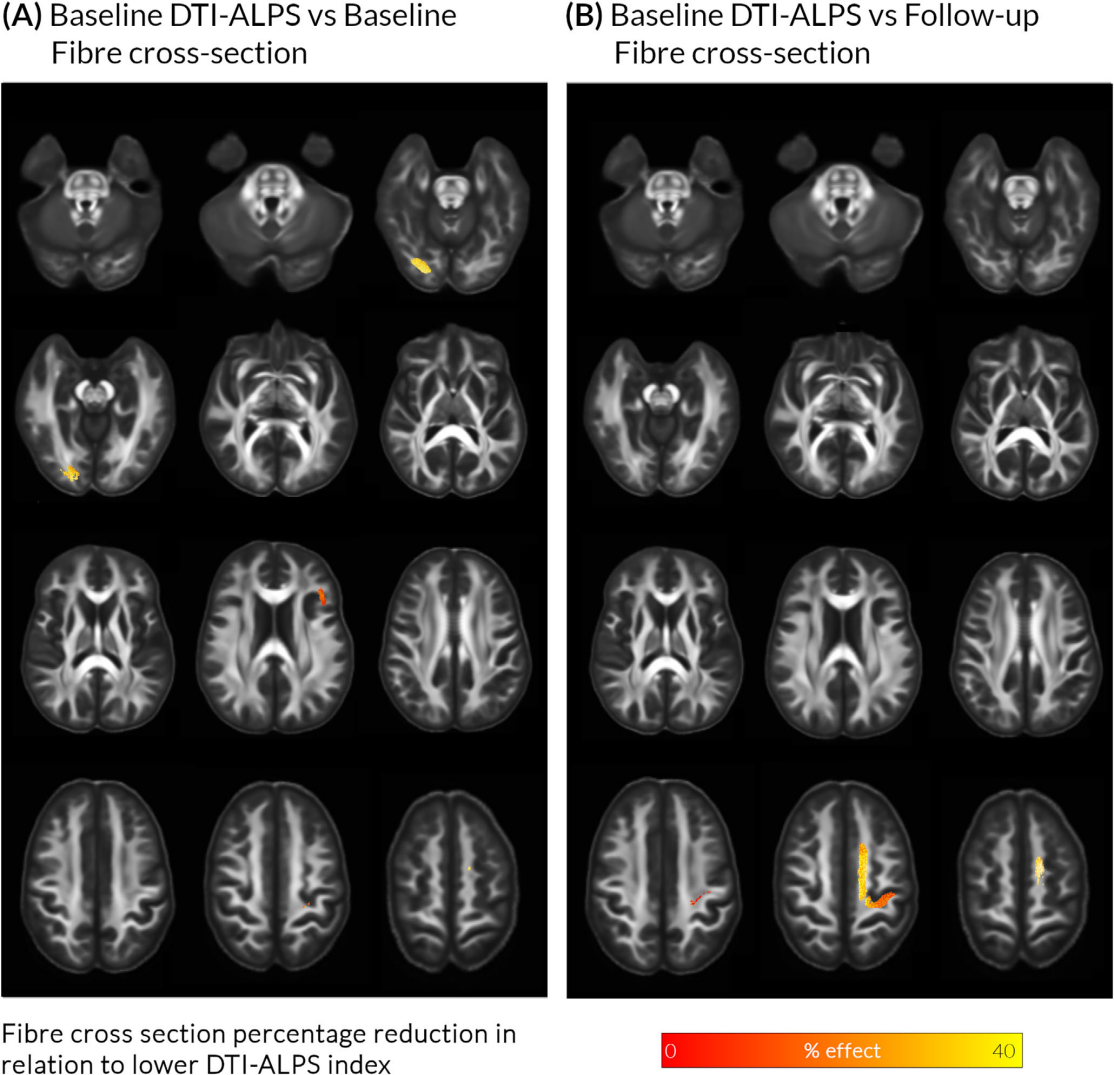
Lower diffusion tensor image analysis along the perivascular space (DTI‐ALPS) index, indicating impaired glymphatic clearance, is associated with white matter macrostructural changes in Parkinson's disease (PD). (A) Baseline reductions in fiber cross‐section in whole‐brain analysis. In patients with PD (n = 98), lower baseline DTI‐ALPS index was associated with reductions in baseline fiber cross‐section, in occipital regions, adjusted for age, sex, and total intracranial volume. (B) Reductions in fiber cross‐section after 3‐year follow‐up (Session 3). In patients with PD (n = 64), lower baseline DTI‐ALPS index was associated with reductions in fiber cross‐section in superior frontal regions, at Session 3, adjusted for age, sex, total intracranial volume, and time between scans. Effect sizes in both cases are shown as percentages and presented as streamlines in template space. Only family‐wise‐error [FWE]‐corrected results are displayed (FWE‐corrected *P* < 0.05). [Color figure can be viewed at wileyonlinelibrary.com]

In whole‐brain analysis, lower DTI‐ALPS index was associated with reduced fiber cross‐section at baseline (*n* = 98, corrected for age, sex, and intracranial volume) within the right optic radiation, bilateral anterior corona radiata, and left arcuate fasciculus (up to 33.6% reduction, FWE‐corrected *q* = 0.007). Lower DTI‐ALPS was associated with reduced fiber cross‐section within left anterior and posterior corona radiata at Session 3 (40.0% reduction, *q* = 0.006). White matter changes associated with DTI‐ALPS are shown in Figure 2; these were spatially distinct from previously described changes linked to cognitive impairment in PD^4^, ^33^ and independent from DTI‐ALPS in mediation analyses (Supplementary Material S1).

Baseline DTI‐ALPS index was not associated with plasma NfL in PD (n = 82) (*β* = −0.351, *P* = 0.448). There was no significant correlation between DTI‐ALPS and *p*‐tau181 (n = 88, *β* = −0.266, *P* = 0.642).

### 2.3 DTI‐ALPS Index is Associated with QSM Signal, Reflecting Brain Iron Redistribution

**FIG. 3:**
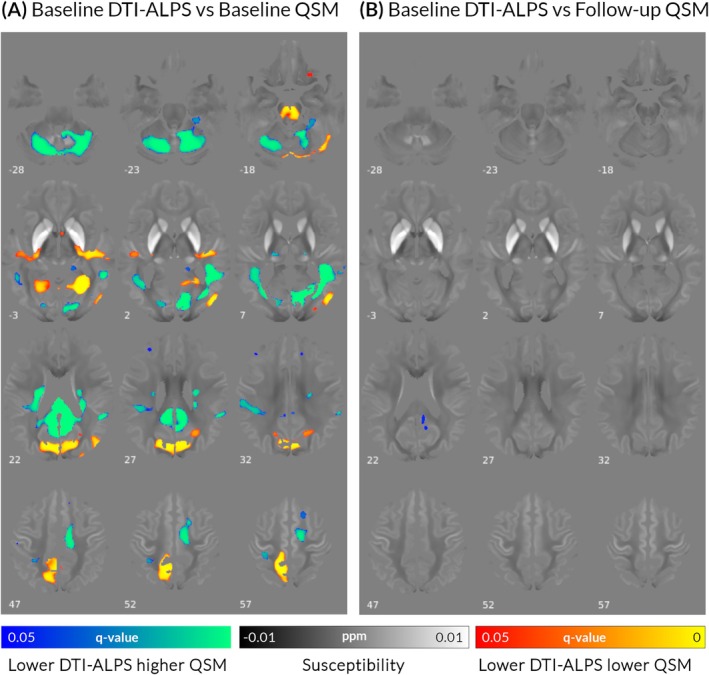
Lower diffusion tensor image analysis along the perivascular space (DTI‐ALPS) index was associated with bidirectional changes in quantitative susceptibility mapping (QSM) levels in patients with Parkinson's disease (PD). (A) Baseline QSM. In patients with PD (n = 98), lower baseline DTI‐ALPS index was associated with both increases (blue colour scale) and reductions (red colour scale) in baseline susceptibility, adjusted for age and sex. (B) Follow‐up increases in QSM. Lower baseline DTI‐ALPS index was associated with increases in susceptibility after 3‐year follow‐up (Session 3) in patients with PD (n = 59), adjusted for age, sex, and time between scans. Results are displayed on the QSM study‐template in MNI152 space; numbers represent axial slice location in MNI152 space. Red/yellow clusters represent voxels where a significant positive correlation was seen with DTI‐ALPS index (family‐wise error [FWE]‐corrected *P*‐value, *q* < 0.05) and blue/green clusters voxels where a significant negative correlation was seen (*q* < 0.05). [Color figure can be viewed at wileyonlinelibrary.com]

For baseline QSM (*n* = 98), lower DTI‐ALPS index was associated with higher QSM signal within bilateral cerebellar peduncles, medial temporal, posterior parietal and frontal lobes, and lower QSM signal within bilateral occipital regions (Fig. 3). Lower baseline DTI‐ALPS was associated with higher QSM signal in right cingulate and posterior parietal lobe after 3 years follow‐up (n = 59, Fig. 3). The spatial distribution of QSM signal change was distinct from previously described changes associated with worse cognition in PD^6^, ^34^ and not mediated by DTI‐ALPS (Supplementary Material S1).

### 2.4 DTI‐ALPS and Cortical Thickness

**FIG. 4:**
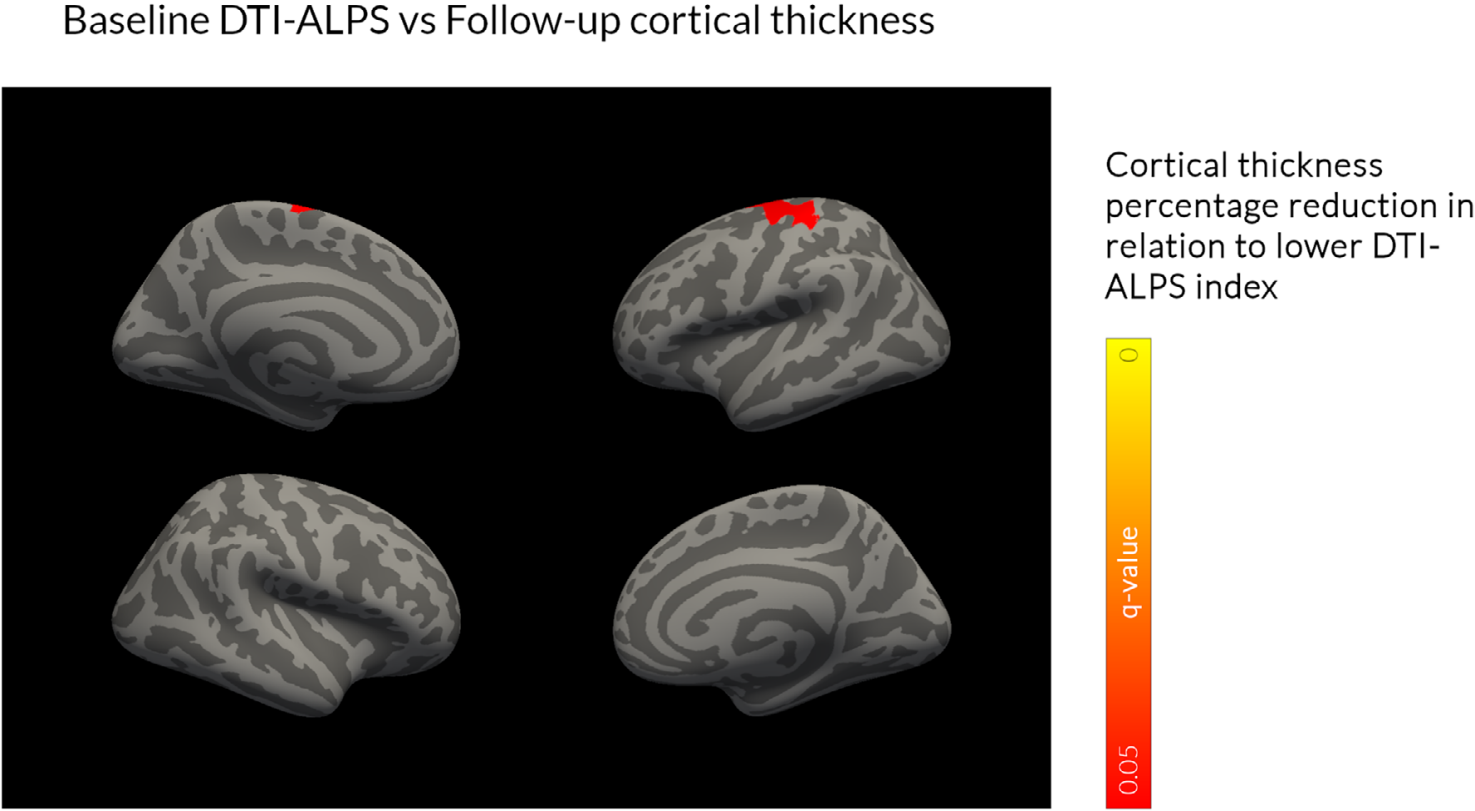
Lower diffusion tensor image analysis along the perivascular space (DTI‐ALPS) index was associated with increased cortical thinning at follow‐up. In patients with Parkinson's disease (PD) there was no correlation between a lower baseline DTI‐ALPS index with baseline cortical thickness (whole‐brain analysis, family‐wise error [FEW]‐corrected *P*‐value, *q* < 0.05, age and sex corrected) but there was an association with percentage reduction in the pre‐central gyrus after 3 years of follow‐up. [Color figure can be viewed at wileyonlinelibrary.com]

At baseline (n = 98) there were no statistically significant correlations in the relationship between DTI‐ALPS index and whole‐brain cortical thickness. Lower baseline DTI‐ALPS was associated with increased longitudinal cortical thinning (annualized percentage reduction) in a cluster within the pre‐central gyrus (n = 57; Fig. 4).

## 3 Discussion

The glymphatic system plays a key role in clearance of pathological proteins and other waste products from the brain and has been postulated to contribute to PD symptoms^23^, ^24^ and cognitive impairment.^28^, ^29^, ^30^ However, how and when glymphatic clearance dysfunction relates to other pathologies that contribute to PD dementia is unclear. Here, we used an indirect, non‐invasive correlate of glymphatic clearance, the DTI‐ALPS index, to examine its relationship with white matter degeneration, iron and amyloid accumulation, and grey matter atrophy. We show that lower DTI‐ALPS index: (1) is associated with cognitive performance and predicts poor outcomes in PD; (2) is associated with white matter macrostructural changes (reductions in fiber cross‐section) and changes in magnetic susceptibility throughout the brain; but (3) its contribution to cognitive decline is distinct to those processes.

First, we show that DTI‐ALPS index is reduced in PD patients who progress to poor outcomes within 3 years, already at baseline, with additional reductions during follow‐up. In contrast, it remained relatively stable in PD patients with stable cognition and controls. The DTI‐ALPS index was significantly associated with cognition and global PD burden. This is in keeping with previous studies showing lower DTI‐ALPS index with worsening global disease severity^23^, ^59^ and cognition^24^ in PD. Other indirect methods of assessing glymphatic clearance also correlate with worse cognition in PD. Higher burden of enlarged perivascular spaces in the basal ganglia correlates to disease severity^23^ and cognitive performance^60^ in PD, and predicts cognitive decline over 5 years.^61^ Glymphatic clearance relies on CSF movement through the perivascular space, and enlargement of perivascular spaces is thought to reflect glymphatic dysfunction.^62^ Polymorphisms of aquaporin‐4, which play a crucial role in glymphatic function, are also linked to different rates of cognitive decline in PD.^63^ Our study adds to the growing body of evidence that impaired glymphatic clearance is linked to cognitive impairment in PD.

Several hypotheses have been postulated as to how impaired glymphatic clearance could contribute to neurodegeneration, mainly focusing on the potential role of the glymphatic system in pathological protein aggregation.^9^, ^64^ Evidence from animal models suggests that suppression of glymphatic clearance leads to accumulation of both alpha‐synuclein^13^ and beta‐amyloid,^15^ whilst misfolded protein spread via the glymphatic system has also been postulated.^9^, ^12^ In addition, lymphatic drainage seems to contribute to iron removal from the central nervous system in rats following intraventricular hemorrhage,^65^ suggesting a possible role in iron clearance; and glymphatic dysfunction may trigger neuroinflammation.^66^ All these processes may contribute in PD dementia: alpha‐synuclein, beta‐amyloid, and tau accumulation, and cerebrovascular changes are seen in PD patients at autopsy.^32^ Increased iron accumulation, particularly within the substantia nigra, is also seen^67^ and may play a role in cell stress and atrophy^68^ and in promoting alpha‐synuclein aggregation.^69^ Neuroinflammatory response contributes to dopaminergic cell loss and neurodegeneration.^70^ These pathological processes lead to structural and functional brain changes and, eventually, cognitive deficits in PD. However, few studies have tried to assess directly how impaired glymphatic clearance contributes to the structural changes seen in PD dementia. Here, we addressed this by assessing the relationship between DTI‐ALPS index and established imaging markers that change in association with cognition in PD.^4^, ^6^, ^33^, ^34^

We found that lower baseline DTI‐ALPS index, an indirect marker of poor glymphatic clearance, is associated with reductions in fiber cross‐section, both at baseline and after 3 years. However, these changes were less diffuse and involved different tracts than those we have previously shown in PD with cognitive impairment using the same methodology^4^, ^33^; for example, strikingly, fiber cross‐section of the corpus callosum, a key area from prior work, was not related to DTI‐ALPS.^4^, ^33^ Additionally, we found no relationship between DTI‐ALPS and plasma NfL, despite the consistent observation of higher plasma NfL in association with cognitive decline in PD.^4^, ^71^, ^72^, ^73^ Another study in genetic frontotemporal dementia failed to find a correlation between plasma NfL and DTI‐ALPS.^74^ A potential explanation is different timing of glymphatic clearance and white matter degeneration. In AD, impaired amyloid clearance predates accumulation and overt neurodegeneration^75^, ^76^; this could also be the case in PD. Glymphatic dysfunction correlates with degree of white matter vascular damage in health, as lower DTI‐ALPS is associated with higher white matter hyperintensity volume at baseline and after 1 year.^77^ In contrast, changes in fiber cross‐section correlate with pathological protein distribution,^50^, ^78^ and NfL is a marker of axonal damage^79^, ^80^ reflecting different mechanisms than white matter hyperintensities.^81^ Therefore, another potential explanation for our findings is that DTI‐ALPS predominantly affects white matter through cerebrovascular changes, which are not captured by fiber cross‐section or NfL. This could be tested in future work specifically examining the relationship between the DTI‐ALPS index and measures of small vessel disease in PD.

We found that lower DTI‐ALPS index was associated with diffuse changes in QSM signal, with both areas of lower and higher magnetic susceptibility at baseline, and these also involved different regions than those previously linked to cognition in PD.^6^, ^34^ Only one prior study has concurrently assessed DTI‐ALPS and magnetic susceptibility: Zhou et al. found a correlation between basal ganglia QSM and DTI‐ALPS adjusting for age and sex in 213 older adults.^31^ This was interpreted as evidence that the glymphatic system plays a role in brain iron accumulation; however, that study was a cross‐sectional association in healthy individuals using region‐of‐interest analysis and did not assess whether these changes were behaviorally relevant. Our findings of bidirectional susceptibility changes in relation to DTI‐ALPS, in keeping with human^31^ and animal evidence,^65^ suggest that glymphatic clearance does indeed play a role in brain iron distribution. However, given the differential spatial distribution and lack of mediating effect, our findings do not support the notion that impaired glymphatic clearance is a direct cause of the iron accumulation seen in PD with cognitive impairment.

Perhaps surprisingly, we did not find a correlation between DTI‐ALPS index and plasma *p*‐tau181. Impaired glymphatic clearance leads to beta‐amyloid and tau accumulation in animal models,^14^, ^15^ and the DTI‐ALPS index is linked to amyloid‐ and tau‐PET positivity in AD.^27^, ^28^ However in PD, plasma *p*‐tau181 levels do not correlate with cognitive severity.^4^, ^72^, ^73^ This could be due to higher heterogeneity of beta‐amyloid and tau pathology in PD which may, in some cases, be incidental rather than driving cognitive decline. Alternatively, other *p*‐tau measurements, such as *p*‐tau217, with increased sensitivity in detecting amyloid pathology,^82^ may have greater ability to identify an association.

We found little evidence for an association between DTI‐ALPS index and cortical thickness in PD: with no relationship at baseline and lower DTI‐ALPS index predicting accelerated cortical thinning only within the precentral gyrus at follow‐up. Previous studies have shown a correlation between DTI‐ALPS index and cortical grey matter volume in AD^28^, ^29^ and cortical thickness in frontotemporal dementia.^83^ However, all studies used a region of interest, rather than a whole‐brain approach, in conditions where cortical atrophy patterns are well‐established and preserved across individuals. In contrast, cortical atrophy is highly heterogeneous in PD, occurring later in the disease course.^25^, ^84^, ^85^, ^86^, ^87^

Across all tested modalities, the spatial profiles of changes in relation to DTI‐ALPS were different to those seen in relation to cognitive decline. This result, confirmed by the lack of mediation effect of DTI‐ALPS to either fiber cross‐section or QSM, suggests that glymphatic clearance does not drive white matter axonal degeneration nor brain iron accumulation in PD, but may play an independent role in cognitive impairment. An alternative explanation is that DTI‐ALPS index is not a robust marker for glymphatic clearance. The DTI‐ALPS index is deductive, and only indirectly reflects glymphatic clearance. It is affected by other factors that influence DWI signal.^88^, ^89^ Additionally, it can only be calculated within a specific region, at the level of the body of the lateral ventricles. Therefore it cannot assess regional differences in glymphatic function.^88^, ^89^ Lastly, it has not been extensively validated in pathophysiological studies. Notwithstanding these limitations, the DTI‐ALPS index is reduced under conditions where glymphatic function is impaired^20^, ^30^ and correlates strongly with intrathecal contrast administration,^18^ the gold‐standard in‐vivo assessment method of glymphatic function. It is therefore likely to reflect glymphatic clearance, albeit not fully or solely. Other non‐invasive methods to assess the glymphatic system are being developed, including perivascular spaces and arterial spin labeling.^88^, ^90^ Future work employing multiple metrics of glymphatic clearance could help elucidate its role in PD dementia.

Our study has some limitations. We did not acquire T_2_/FLAIR (fluid‐attenuated inversion recovery) imaging and therefore could not quantify small vessel disease. Small vessel disease relates to cognition in PD^91^, ^92^ and negatively correlates with DTI‐ALPS^77^, ^93^; it is possible that it mediates DTI‐ALPS' effect on cognition in PD, and this could be examined in future work. Both DTI‐ALPS and fiber cross‐section are derived from DWI, albeit using different computational models. We attempted to minimize bias within specific regions used in DTI‐ALPS calculation using an interhemispheric approach but future studies should examine how white matter changes influence DTI‐ALPS calculations. Although ours was a longitudinal cohort, only group‐level effects can be examined in our study design, and whether DTI‐ALPS is useful at the individual level remains to be determined. Additionally, this was a single‐center study and findings should be validated in additional populations. Finally, we were not adequately powered to investigate subgroup‐specific relationships between DTI‐ALPS and whole‐brain imaging metrics.

## 4 Conclusions

The DTI‐ALPS index is lower in PD patients who progress to poor outcomes over 3 years and correlates with cognition. We show that although the DTI‐ALPS index is linked to poor cognitive outcomes in PD, it is not the driver of the white matter axonal degeneration, or the iron accumulation seen in PD patients with cognitive impairment. Whether this reflects limitation of the technique or an independent role of impaired glymphatic clearance in Parkinson's dementia remains to be seen.

## Author Roles

(1) Research Project: A. Design, B. Execution, C. Analysis; (2) Statistical Analysis: A. Design, B. Execution, C. Review and Critique; (3) Manuscript Preparation: A. Writing of the First Draft, B. Review and Critique.

A.Z.: 1A, 1B, 1C, 2A, 2B, 2C, 3A, 3B.

G.T.: 1C, 2C, 3B.

R.P.: 2C, 3B.

N.H.: 1B, 2B, 2C, 3B.

I.D.: 1B, 2B, 2C, 3B.

A.J.H.: 1B, 2B, 3B.

E.V.: 1B, 2B, 3B.

H.Z.: 1B, 2C, 3B.

R.S.W.: 1A, 2A, 2C, 3A, 3B.

## Supporting information


**Data S1.** Supporting Information.

**Data S2.** STROBE (Strengthening the Reporting of Observational Studies in Epidemiology) statement: reporting guidelines checklist for cohort, case–control, and cross‐sectional studies.

## Data Availability

The data that support the findings of this study are available on request from the corresponding author. The data are not publicly available due to privacy or ethical restrictions.
