# Supplementary material for: Impaired Glymphatic Clearance, Measured Using Diffusion Tensor Image Analysis Along the Perivascular Space (DTI‐ALPS), is Linked to Poor Cognitive Outcomes in Parkinson's Disease
Source: Mov Disord. 2025 Aug 7;40(11):2367–80. doi: 10.1002/mds.30325 (PMC12661622; doi:10.1002/mds.30325)
Supplement: Supplementary file 1 — Data S1. Supporting Information. [file MDS-40-2367-s002.docx]

# Supplementary Material:

# Impaired glymphatic clearance independently contributes to poor outcomes in Parkinson’s

Dr Angeliki Zarkali PhD^1,2^, Dr George Thomas PhD^1^, Dr Ross Paterson PhD, MBBS^1,2^, Ms Naomi Hannaway MSc^1^, Ms Ivelina Dobreva MSc^1^, Dr Amanda J Heslegrave PhD^3^, Dr Elena Veleva PhD^3^, Prof Henrik Zetterberg PhD^1,3^, Prof Rimona S Weil PhD^1,2,4,5^

## **S1. Replication of DTI-ALPS findings only in Parkinson’s (PD) patients with dementia or mild cognitive impairment (n=27), PD patients with intact cognition (n=67) and controls (n=28)**


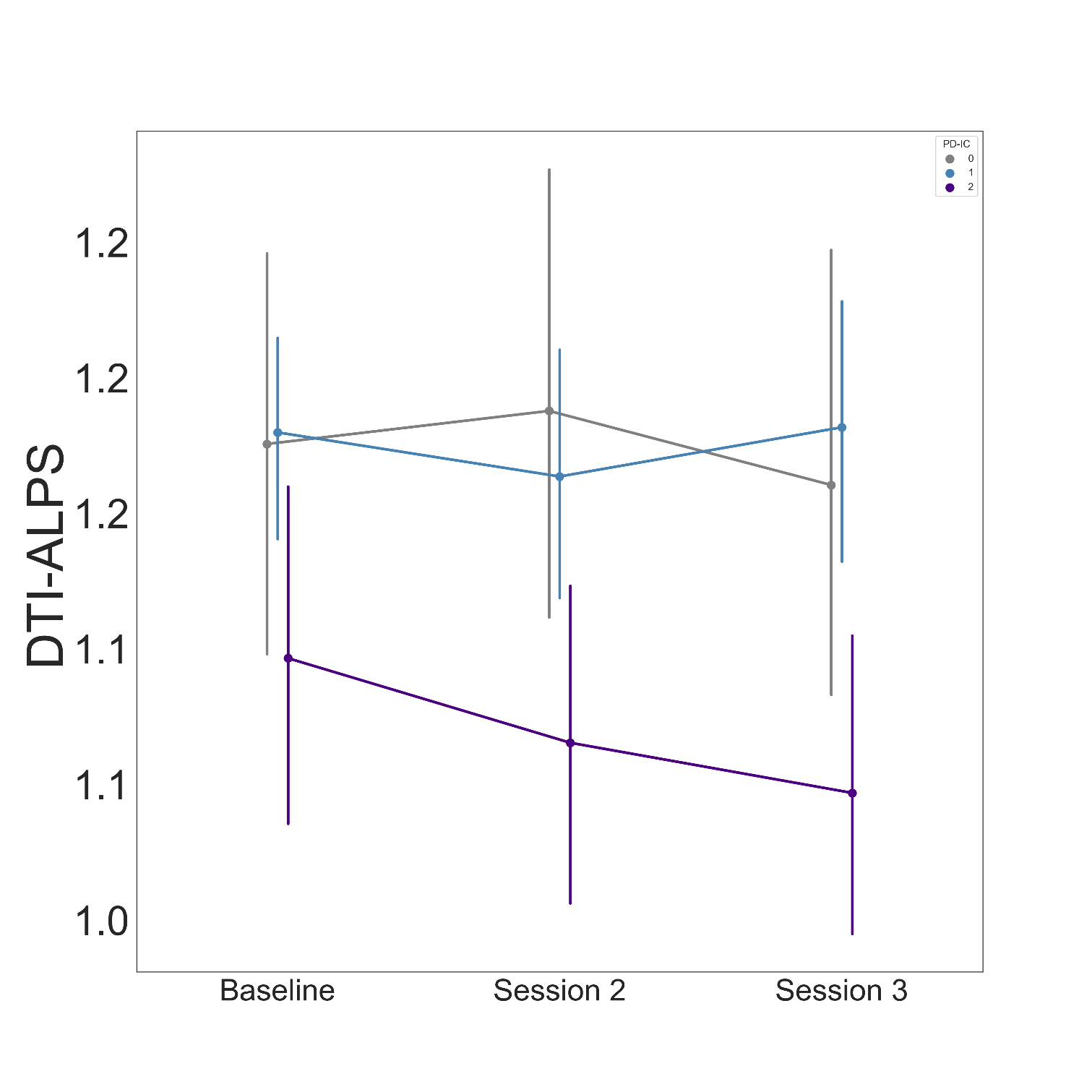


Controls

PD normal cognition

PD impaired cognition

***Figure S1. DTI-ALPS index is lower in PD patients with impaired cognition compared to those with intact cognition, and worsens over follow-up***

*Patients with Parkinson’s who developed dementia or mild cognitive impairment had lower DTI-ALPS index at baseline than PD with intact cognition(p<0.001) and controls (p=0.002). They also showed additional longitudinal reductions (group*time interaction β=-0.014, p=0.017) during follow-up. DTI-ALPS: diffusion tensor image analysis along the perivascular space.*

## **S2. Mediation analyses between glymphatic function (DTI-ALPS index), white matter degeneration (fibre cross-section), grey matter atrophy (cortical thickness), iron accumulation (QSM) and cognitive outcomes in Parkinson’s disease**

**Methods**

To explore the interplay between glymphatic function, white matter degeneration, iron accumulation and poor cognitive outcomes in Parkinson’s, we conducted a mediation analysis to derive total, direct and indirect effects. To perform mediation analysis, mean fibre cross-section, grey matter volume and quantitative susceptibility mapping (QSM) values were derived from regions of interest (ROIs) that were most related to cognition based on our previous work in the same population cohort (Thomas et al., 2020; Zarkali et al., 2024, 2021): the corpus callosum for white matter, hippocampus for grey matter and nucleus basalis of Meynert (NBM) for QSM. Mean fibre cross-section was derived from the entirety of the corpus callosum using a mask from the JHU white matter atlas. Mean bilateral hippocampal volume was derived from the built-in Freesurfer segmentation. Mean signed QSM was derived from a manually traced mask of the NBM as previously described (Thomas et al., 2020).

We evaluated the inter-relationship between QSM, fibre cross-section, hippocampal volume and DTI-ALPS using partial correlation with age and sex as covariates. Additionally, we assessed each variable’s relationship with 1) change in combined cognitive scores (Session 3 – Baseline) and 2) poor outcomes. Only variables that were correlated both with each other and with the outcome of interest (change in combined scores or poor outcomes respectively) were included in subsequent mediation analyses (*Table S1*).

Mediation analysis was then performed between change in 1) combined cognitive scores (Session 3 – Baseline) and baseline mean QSM and DTI-ALPS, with age and sex as covariates using linear regression and 2) poor outcomes and hippocampal volume and corpus callosum fibre cross-section using logistic regression.

**Results**

QSM signal of the NBM had a direct effect on change in cognitive scores (β=-4.445, p=0.001) (*Figure A*). Neither QSM nor DTI-ALPS acted as a mediator for the other, suggesting independent contributions to cognitive decline. Mean corpus callosum fibre cross-section had a significant direct effect on poor outcomes (β=1.348, p=0.01) and a small effect on cortical thickness (β=0.001, p<0.001) (*Figure B*). DTI-ALPS was not correlated with corpus callosum fibre cross-section (rho= -0.051, p=0.673) or hippocampal volume (rho=-0.183, p=0.129).

***Table S1. Univariate correlation between two different cognitive outcome measures, DTI-ALPS index and other imaging metrics***

| **Factor** | **Change in combined cognitive scores (Session 3 – Session 1)** | |
| --- | --- | --- |
|  | **rho** | **p** |
| DTI – ALPS | **-0.111** | **0.038** |
| QSM | **0.272** | **<0.001** |
| WM | -0.113 | 0.310 |
| GM | -0.116 | 0.342 |
| **Factor** | **Poor cognitive outcomes** | |
| DTI – ALPS | -0.053 | 0.661 |
| QSM | -0.147 | 0.173 |
| WM | **-0.240** | **0.019** |
| GM | **-0.257** | **0.032** |
| *Partial spearman correlation with age and sex as covariates.*  *DTI-ALPS: diffusion tensor imaging along the perivascular space, GM: mean bilateral hippocampal volume, QSM: mean quantitative susceptibility mapping signal within the nucleus basalis of Meynert, WM: mean fibre cross section of the corpus callosum* | | |


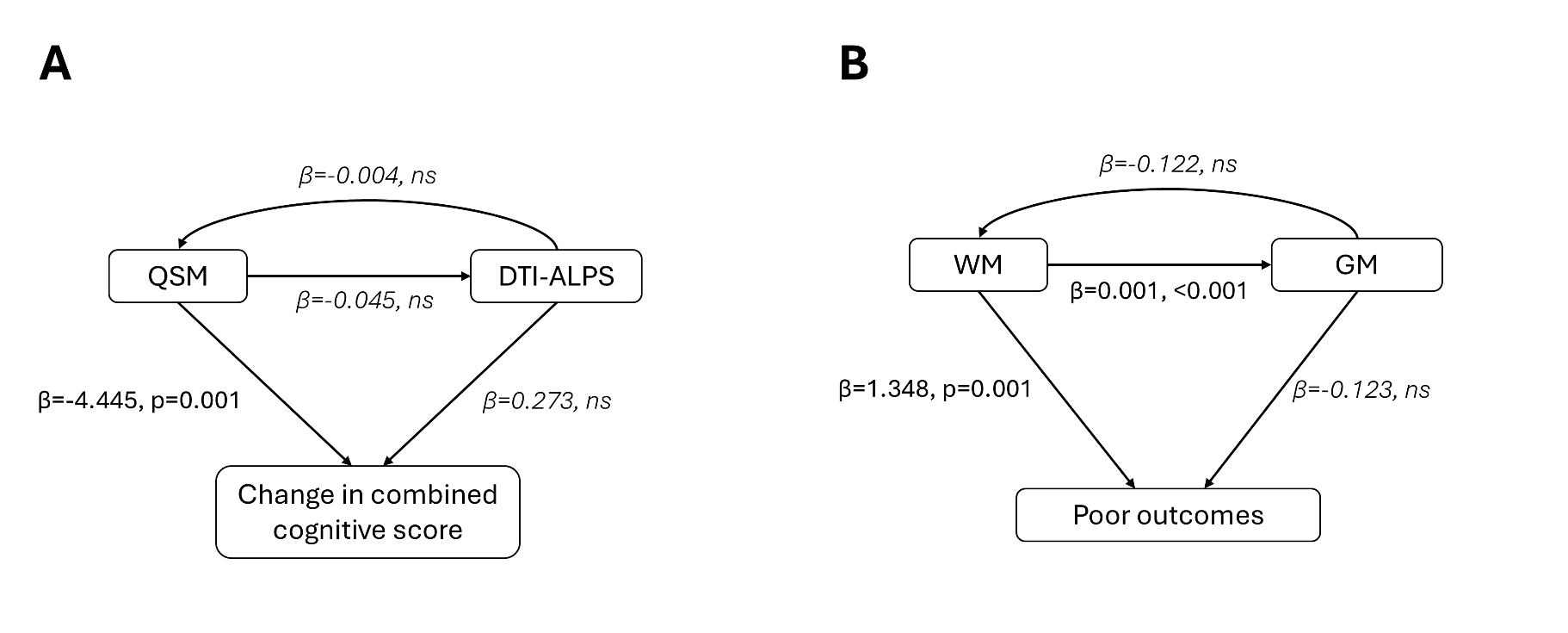
***Figure S2. Mediation analysis of factors contributing to cognitive change in Parkinson’s.***

*Standardized coefficients (β) were calculated for each association with (****A****) change in combined cognitive scores (Session 3 – Baseline) and (****B****) poor outcomes in patients with Parkinson’s disease, using mediation analysis of baseline values for each variable adjusted for age and sex.*

*DTI-ALPS: diffusion tensor image analysis along the perivascular space; GM: bilateral hippocampal volume; ns: non-significant (p-value>0.05); QSM: mean quantitative susceptibility mapping value of the nucleus basalis of Meynert; WM: mean fibre cross-section of the corpus callosum.*
